# Metformin Increases Protein Phosphatase 2A Activity in Primary Human Skeletal Muscle Cells Derived from Lean Healthy Participants

**DOI:** 10.1155/2021/9979234

**Published:** 2021-07-28

**Authors:** Aktham Mestareehi, Xiangmin Zhang, Berhane Seyoum, Zaher Msallaty, Abdullah Mallisho, Kyle Jon Burghardt, Anjaneyulu Kowluru, Zhengping Yi

**Affiliations:** ^1^Department of Pharmaceutical Sciences, College of Pharmacy and Health Sciences, Wayne State University, Detroit, MI 48201, USA; ^2^Division of Endocrinology, Wayne State University School of Medicine, Wayne State University, Detroit, MI 48201, USA; ^3^Department of Pharmacy Practice, Eugene Applebaum College of Pharmacy/Health Sciences, Wayne State University, Detroit, MI, USA; ^4^Program for Translational Research in Diabetes, Biomedical Research Service, John D. Dingell VA Medical Center, Detroit, MI 48201, USA

## Abstract

**Objective:**

To investigate if PP2A plays a role in metformin-induced insulin sensitivity improvement in human skeletal muscle cells. *Participants*. Eight lean insulin-sensitive nondiabetic participants (4 females and 4 males; age: 21.0 ± 1.0 years; BMI: 22.0 ± 0.7 kg/m^2^; 2-hour OGTT: 97.0 ± 6.0 mg/dl; HbA1c: 5.3 ± 0.1%; fasting plasma glucose: 87.0 ± 2.0 mg/dl; *M* value; 11.0 ± 1.0 mg/kgBW/min).

**Design:**

A hyperinsulinemic-euglycemic clamp was performed to assess insulin sensitivity in human subjects, and skeletal muscle biopsy samples were obtained. Primary human skeletal muscle cells (shown to retain metabolic characteristics of donors) were cultured from these muscle biopsies that included 8 lean insulin-sensitive participants. Cultured cells were expanded, differentiated into myotubes, and treated with 50 *μ*M metformin for 24 hours before harvesting. PP2Ac activity was measured by a phosphatase activity assay kit (Millipore) according to the manufacturer's protocol.

**Results:**

The results indicated that metformin significantly increased the activity of PP2A in the myotubes for all 8 lean insulin-sensitive nondiabetic participants, and the average fold increase is 1.54 ± 0.11 (*P* < 0.001).

**Conclusions:**

These results provided the first evidence that metformin can activate PP2A in human skeletal muscle cells derived from lean healthy insulin-sensitive participants and may help to understand metformin's action in skeletal muscle in humans.

## 1. Introduction

In the US, ∼30 million individuals have type 2 diabetes (T2D), which is associated with long-term damage of various organs (e.g., eyes, kidneys, nerves, heart, and brain). Insulin resistance in skeletal muscle is one of the main causes for T2D [1]. Metformin (N,N-dimethylbiguanide) is an effective oral biguanide antihyperglycemic drug and the most frequently prescribed as a first-line therapy for T2D [2]. Metformin reduces glucose production by the liver and increases insulin sensitivity (i.e., decreased insulin resistance) in skeletal muscles [2, 3]. In addition, links between insulin resistance, diabetes, and brain disorders have been reported [4, 5], and metformin has been shown to have antiseizure effects in epilepsy animal models and is proposed as a potential candidate for drug repurposing for epilepsy patients [5–7]. Nonetheless, molecular mechanisms of metformin's action in skeletal muscle are incompletely understood. Protein phosphatase 2A (PP2A) is a ubiquitously expressed serine/threonine phosphatase and plays a pivotal role in cellular processes, such as insulin signal transduction, cell proliferation, and apoptosis, by dephosphorylating key signaling molecules such as AKT, AMPK, p53, and c-Myc [8]. Metformin may activate PP2A [9, 10], while others have shown that metformin has no effect on PP2A [11]. Whether metformin affects PP2A activity in primary human skeletal muscle cells remains to be elucidated. The overall objective of this study was to determine whether metformin activates PP2A in primary human skeletal muscle cells.

## 2. Materials and Methods

### 2.1. Antibodies and Reagents

The PP2A activity assay kit was purchased from EMD Millipore (cat. 17313), which has been successfully used in various studies [12–14]. The PP2A activity assay kit contains protein A agarose, anti-PP2A, C subunit, clone 1D6 (catalog # 05-421), normal mouse IgG (catalog # 12-371), protein A agarose (catalog # 16-125D), threonine phosphopeptide (K-R-pT-I-R-R) (catalog # 12-219), pNPP Ser/Thr assay buffer (catalog # 20-179), Malachite green additive (solution A & B) (catalog # 20-105 and catalog # 20-104), phosphate standard (solution C) (catalog # 20-103), and 96-well microtiter plate (½ volume flat bottom plate). Metformin hydrochloride (Tocris Bioscience, Bristol, UK) was prediluted in purified water as ×10 stock and preserved under −80°C before use. Bradford reagent was purchased from Sigma.

### 2.2. Experimental Design

The overall experimental design is presented in Supplementary Figure 1. Briefly, extensive subject recruitment was followed by comprehensive screening tests (e.g., vitals, 2-hour oral glucose tolerance test (2 h OGTT), and blood chemistry). Eligible subjects underwent in-patient clinical tests: a skeletal muscle biopsy followed by a 2-hour hyperinsulinemic-euglycemic clamp to assess insulin sensitivity. Biopsies were immediately blotted free of blood and cleaned of connective tissue (~30 sec), submerged in ice-cold DPBS for primary skeletal muscle cell culturing as described in references [15–18]. The primary cells were differentiated into myotubes and treated with/out metformin. The cells are lysed, and protein concentration was measured. Two hundred *μ*g lysate proteins from each sample were utilized for PP2A activity assay. NIgG immunoprecipitation was used to determine the background phosphate activity, and the supernatant from the NIgG immunoprecipitation was subjected to PP2Ac immunoprecipitation. Both NIgG and PP2Ac immunoprecipitates were incubated with the phosphatase substrate (i.e., threonine phosphopeptide (K-R-pT-I-R-R)), and the resulting supernatants were mixed with Malachite green phosphate detection solution, followed by the measurement of the absorbance at 650 nm. The amount of free phosphate released (reflecting phosphatase activity) was calculated from a standard curve. The resulting PP2A activity data were integrated with clinical data.

### 2.3. The Clinical Studies and Subjects

This protocol was approved by the Institutional Review Board of Wayne State University. The clinical studies started with participant recruitment, followed by comprehensive screening tests (Visit 1) and involved hyperinsulinemic-euglycemic clamp and muscle biopsies (Visit 2). All participants were instructed to stop any form of exercise for at least 2 days before each visit, and none of them had any significant medical problems. On Visit 1 day, the subject arrived at the clinical research service center in the morning after a 10-hour overnight fast. Written consent was obtained before their participation, and the purpose and potential risks of the study were explained to all participants. Measurement of vitals, urine analysis, 2-hour oral glucose tolerance test (OGTT), pregnancy test if female participant, and 12-lead electrocardiogram (ECG) were performed at site. HbA1c and blood chemistry were measured by the Detroit medical center (DMC). Eligible participants (a total of 8 volunteers) were scheduled for Visit 2.

### 2.4. Muscle Biopsy and Hyperinsulinemic-Euglycemic Clamp

The hyperinsulinemic-euglycemic clamp is considered to be the gold standard for measuring insulin sensitivity in vivo as previously described [18–20]. The study began at approximately 8 am (time −60 min) after an overnight fast. A catheter was placed in an antecubital vein and maintained throughout the study for infusions of insulin and glucose. A second catheter was placed in a vein in the contralateral arm for the sampling of arterialized venous blood. Blood glucose was measured and reported every 15 minutes before insulin infusion started and every 5 minutes after insulin infusion started. Around 8:30 am (time −30 min), a licensed physician performed the muscle biopsies from the vastus lateralis using the modified Bergstrom technique under local anesthesia (lidocaine) [21]. The collected biopsies were immediately blotted free of blood and cleaned of connective tissue and fat (~30 sec) and submerged in ice-cold media and transported to the laboratory for primary skeletal muscle cell culturing as described previously [15–17]. At ~9 am (time 0 min), a primed, continuous infusion of human regular insulin (Humulin R; Eli Lilly, Indianapolis, IN) was initiated and continued for 120 minutes to quantify the effects of insulin on glucose disposal. Insulin infusion was started at 160 mU/(m^2^·min) for 5 minutes, followed by 120 mU/(m^2^·min) for 5 minutes, and then maintained at constant 80 mU/(m^2^·min). This ensured that steady-state levels were more rapidly achieved, minimizing the burden on the participants. Throughout the insulin infusion, an infusion of 20% glucose was adjusted to maintain euglycemia (targeted at 90 mg/dl). Insulin-stimulated glucose disposal rate (*M* value) was calculated as the average glucose infusion rate value during the final 30 min of insulin infusion.

### 2.5. Primary Cell Cultures and Treatments

The biopsy was washed 3x in ice-cold phosphate buffer saline (PBS) and minced into small pieces with 0.05% Trypsin-EDTA added. The minced tissues were centrifuged and filtered through a nylon mesh. The resulting human skeletal muscle cells were cultured in growth medium (Dulbecco modified Eagle's medium) supplemented with 10% FBS, 1% PSG, 10 ng/ml EGF, 0.4 *μ*g/ml dexamethasone, 50 *μ*g/ml fetuin, 1% sodium pyruvate, and 1% NEAA and maintained in a humidified atmosphere at 37°C and 5% CO_2_. The human skeletal muscle cells expanded, and the culture medium was completely changed every other day. The myoblasts were differentiated into myotubes and treated with or without 50 *μ*M metformin for 24 hours as described in the Supplementary Materials.

### 2.6. PP2A Activity Assay

The PP2A activity was measured according to the manufacturer's protocol. The cells were harvested and homogenized in 1 ml of lysis activity buffer included (20 mM imidazole-HCl, 2 mM EDTA, 2 mM EGTA, pH 7.0, with 10 *μ*g/ml each of aprotinin, leupeptin, 1 mM benzamidine, and 1 mM PMSF). The cells were collected in Eppendorf tubes, homogenized for 10 minutes at 4°C, and centrifuged at 11000 × g for 15 minutes at 4°C. The protein concentration was measured using Bradford protein assay method. 200 *μ*g of total protein of each sample was transferred to new Eppendorf tubes, including 30 *μ*l of protein A agarose and 4 *μ*l of normal mouse IgG, and the volumes were topped off to 1000 *μ*l with pNPP assay buffer. The NIgG immunoprecipitates were incubated for one and half hour at 4°C with constant rocking and followed by centrifugation at 3000 × g for 3 minutes at 4°C. The supernatants were transferred to new Eppendorf tubes, including 30 *μ*l of protein A agarose and 4 *μ*l of anti-PP2Ac antibody, and the volumes were topped off to 1000 *μ*l with pNPP assay buffer. All tubes were incubated at constant rotated for 2 hours at 4°C and centrifuged at 3000 × g for 3 minutes at 4°C. The immunoprecipitated PP2Ac and NIgG beads were washed 3 times with 700 *μ*l Tris-buffered saline (TBS) (3000 × g for 3 minutes at 4°C), once with 500 *μ*l pNPP Ser/Thr assay buffer, followed by incubation with 60 *μ*l of 750 *μ*M of threonine phosphopeptide (K-R-pT-I-R-R) and 20 *μ*l of pNPP Ser/Thr assay buffer at 30°C for 10 minutes in a shaking incubator. The beads were centrifuged 3000 × g for 3 minutes at 4°C, and 25 *μ*l of the supernatants was mixed with 100 *μ*l of Malachite green phosphate detection solution into each well to a 96-well plate microtiter plate to terminate the reaction, and the color was allowed to develop for 15 minutes at room temperature. The absorbance of the samples was read using a microplate reader at 650 nm. The amount of free phosphate released was calculated from a standard curve (according to the manufacturer's protocol) and normalized to that of the controls.

### 2.7. Statistical Analysis

All data were represented as the mean ± standard error of the mean (SEM) from eight independent experiments. The significances between the control and metformin-treated groups were analyzed by independent Student's *t*-test.

## 3. Results

We enrolled 8 lean healthy human subjects. A hyperinsulinemic-euglycemic clamp was performed to assess insulin sensitivity, and skeletal muscle biopsy samples were obtained. As can be seen from Supplementary Table 1, these participants are healthy, without family history of T2D, and they are nondiabetic, non-prediabetic with normal HbA1c, normal fasting plasma glucose, normal glucose tolerant, and high insulin sensitivity.

**Figure 1:**
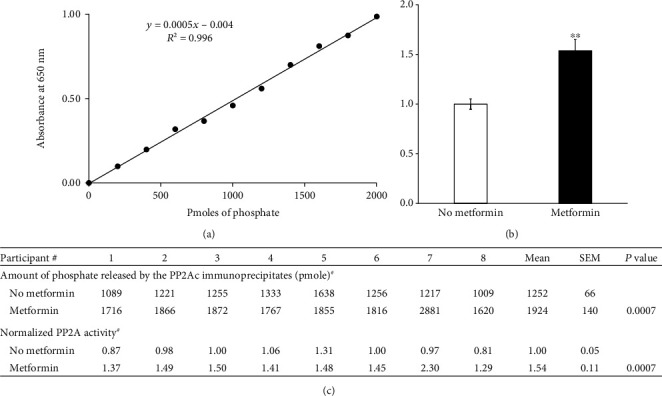
Effect of metformin on PP2A activity in primary human muscle cells derived from 8 lean insulin-sensitive nondiabetic participants (*n* = 8). (a) Measurement of phosphate concentration of the standard solutions (according to the manufacturer's protocol). (b) Summative PP2A activity. Data are given as fold changes (means ± SEM). The mean of the PP2A activity for the 8 samples without metformin treatment was set to 1.00, and the fold changes were relative to basal (i.e., no metformin treatment). (c) Individual PP2A activity. The significance between the basal and metformin-treated groups was analyzed by independent Student's *t*-test. ^∗∗^*P* < 0.001. ^#^Amount of phosphate released reflects the PP2A activity, and the mean of the PP2A activity for the 8 samples without metformin treatment was set to 1.00.

Primary human skeletal muscle cells were cultured from these muscle biopsies, expanded, differentiated into myotubes, and treated with/without 50 *μ*M metformin for 24 hours. Myotubes were homogenized in phosphate free buffer, followed by PP2A activity assay. A standard curve was generated using the standard phosphopeptide (Figure 1(a) with *R*^2^ at 0.996). The slope and intercept were used to calculate the amount of phosphate released by the PP2Ac immunoprecipitates incubated with the standard phosphopeptide. Negligible PP2Ac activity in the NIgG immunoprecipitation samples was detected, indicating our assays were not contaminated with exogenous phosphate or nonspecific immunoprecipitates. PP2A activity measurement indicated that metformin increased the activity of PP2A in the myotubes derived from all eight lean nondiabetic participants as presented in Figures 1(b) and 1(c) (fold increase metformin/basal: 1.54 ± 0.11, *P* < 0.001). These results provided the first evidence that metformin promotes activation of PP2A in human skeletal muscle cells derived from lean, insulin-sensitive, nondiabetic participants.

Correlation analysis of PP2A activity (either with or without metformin treatment) with participants' clinical characteristics indicated that PP2A activity has significant correlation with none of the clinical characteristics for the 8 lean healthy participants except for the heart rate (Supplementary Table 2). One possible explanation is that the participants in this study were homogeneous and all of them are lean, young, and healthy with high insulin sensitivity. Whether PP2A activity has significant correlations with clinical characteristics in obese insulin-resistant participants or T2D patients warrants further investigation.

## 4. Discussion

The molecular mechanisms of metformin are not fully understood. Metformin inhibits complex I in the mitochondrial electron transport chain in liver and muscle cells as well as skeletal muscle [22]. Another potential molecular mechanism is the activation of AMPK in skeletal muscle after metformin treatment [3].

Multiple studies have reported the effect of metformin on PP2A activity. Deepa et al. reported that metformin has no effect on PP2A activity in C2C12 myotube (a mouse cell line) [11]. On the other hand, compatible with our results, several studies have demonstrated that metformin induced PP2A activity in several cell systems. For example, in lung cancer cells (A549 and H1651), metformin-activated PP2A reduced tumor formation *in vivo* and decreased tumor cell growth and invasion capacity *in vitro* as well as serine phosphorylation level of Bax (Ser184), Myc (Ser62), and Akt (Ser473) [23]. Sacco et al. reported that metformin treatment induced the protein expression of PP2A catalytic and regulatory subunit in breast cancer cells in vitro [24]. Notably, Auger et al. have reported that metformin activated PP2A in human subcutaneous white adipose tissue (scWAT), resulting in dephosphorylation of acetyl-CoA carboxylase (Ser79) and hormone-sensitive lipase (Ser660), and metformin also lowered lipolysis in beige fat [25]. Kawashima et al. demonstrated that metformin treatment activated PP2A in myeloproliferative neoplasm (MPN) cells to suppress the oncogenic kinase JAK2V617F by increasing reactive oxygen species levels leading to the inhibition of SHP-2, a positive regulator of JAK2V617F; furthermore, the results indicated that metformin suppressed phosphorylation of the PP2Ac at Tyr307 in a dose-dependent manner. These results revealed that metformin activated PP2A independent of AMPK activation [26]. PP2A is one of the major tau phosphatases, and metformin increased PP2A activity and reduced tau phosphorylation at PP2A-dependent epitopes in vitro and in vivo from primary cortical neuron cells [10]. Metformin treatments induced PRKN gene transcription, mitochondria integrity, mitophagy, and cell viability and decreased activation of nuclear factor kappa B (NF-*κ*B) but not of p53 or ATF4 in human renal epithelial cells [27]. Zhang et al. reported that metformin decreased NLRP3 protein expression and NLRP3 inflammasome activation in ox-LDL-stimulated macrophages through AMPK and PP2A. PP2A catalytic activity was required for NF-*κ*B inhibition and tristetraprolin activation induced by metformin in ox-LDL-stimulated macrophages [9].

Despite the evidence, albeit indirect, on potential regulation of protein phosphorylation-dephosphorylation of key cellular proteins and functions by metformin, putative mechanisms underlying metformin-mediated activation of PP2A remain unclear at this time. PP2A is regulated by the mTOR kinase, and both enzymes affect the phosphorylation status of the ribosomal protein S6 and p70S6K. PP2A has been shown to dephosphorylate the mTOR substrate S6K1 and involved in mTOR-mediated phosphorylation of insulin receptor substrate 1 through modulation of PP2A activity by mTOR [28]. Along these lines, Kickstein and coworkers have reported that metformin directly affected PP2A independently of AMPK/TOR signaling [10]. Based on the data accrued in these studies, the authors have proposed that metformin interferes with association of PP2Ac with MID1-*α*4 complex leading to regulation of PP2A activity. On the other hand, activation or phosphorylation of AMPK by metformin leads to the inhibition of mTOR singling and subsequent dissociation of PP2A and *α*4 and resulting in PP2A activation [29]. It has also been proposed that metformin could dissociate the binding of PP2A and MID1 resulting in decreased PP2A degradation and enhanced phosphatase activity [10, 29]. Additionally, it is likely that metformin could promote interaction between the structural (A subunit), regulatory (B subunit), and catalytic C subunits leading to functional activation of the enzyme. Such interactions may be influenced by posttranslational modification of individual subunits, including the methylation of the catalytic subunit at Leucine-309 residue [8], which has been shown to promote PP2A activation. Future studies will address these aspects to provide mechanical insights into metformin-induced PP2A activation that we report in the current study.

## 5. Conclusions

In the present study, we demonstrated that metformin significantly increased PP2A activity in the myotubes derived from all eight lean nondiabetic participants with high insulin sensitivity. These results provided the first evidence to suggest that metformin promotes activation of PP2A in human skeletal muscle cells and might aid potential new targets for novel mechanistic studies on skeletal muscle insulin resistance in humans.

## Data Availability

The Supplementary Tables data used to support the findings of this study are included within the supplementary information file.

## Abbreviations

AMPK: AMP-activated protein kinase
AKT: Serine/threonine protein kinase B (PKB)
mTOR: Mammalian target of rapamycin
PP2A: Serine/threonine protein phosphatase 2A
NIgG: Normal mouse IgG
PP2Ac: PP2A catalytic subunit
MPN: Myeloproliferative neoplasm
NF-*κ*B: Nuclear factor kappa B
EDTA: Ethylenediaminetetraacetic acid
FBS: Fetal bovine serum
EGTA: Ethylene glycol bis (2-aminoethyl ether)-N,N,N′N′tetraacetic acid.

## Supplementary Materials

Supplementary Materials Supplementary information includes Supplementary Figure 1, Supplementary Table 1, and Supplementary Table 2 as well as supplementary method. Supplementary 1 Supplementary Table 1: clinical characteristics of lean nondiabetic participants in the study. All measurements were done after an overnight fast. Supplementary Table 2: correlation of PP2A activity (either with or without metformin treatment) with participants' clinical characteristics. Supplementary 2 Supplementary Figure 1: schematic diagram of clinical and biological studies.
