# Supplementary material for: Metformin Increases Protein Phosphatase 2A Activity in Primary Human Skeletal Muscle Cells Derived from Lean Healthy Participants
Source: J Diabetes Res. 2021 Jul 28;2021:9979234. doi: 10.1155/2021/9979234 (PMC8342103; doi:10.1155/2021/9979234)
Supplement: Supplementary Materials — Supplementary information includes Supplementary Figure 1, Supplementary Table 1, and Supplementary Table 2 as well as supplementary method. Supplementary 1 Supplementary Table 1: clinical characteristics of lean nondiabetic participants in the study. All measurements were done after an overnight fast. Supplementary Table 2: correlation of PP2A activity (either with or without metformin treatment) with participants' clinical characteristics. Supplementary 2 Supplementary Figure 1: schematic diagram of clinical and biological studies. [file 9979234.f1.zip › 9979234.f1/Supplementary material_20210706.docx]

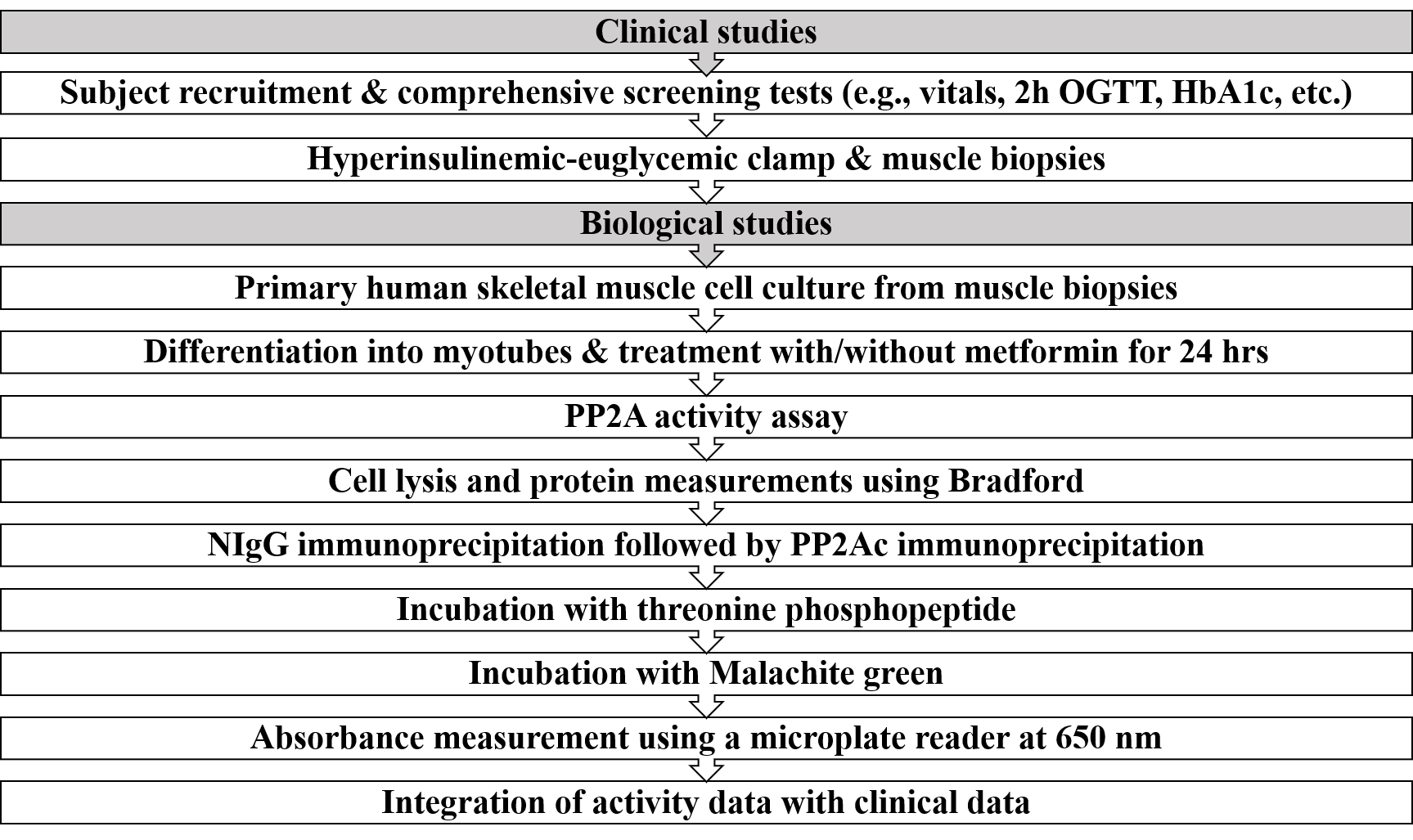


**Supplementary Figure 1.** Schematic diagram of clinical and biological studies.

**Supplementary Table 1.** Clinical characteristics of lean non-diabetic participants in the study. All measurements were done after an overnight fast.

| Participant # | 1 | 2 | 3 | 4 | 5 | 6 | 7 | 8 | **Mean** | **SEM** |
| --- | --- | --- | --- | --- | --- | --- | --- | --- | --- | --- |
| Gender (M/F) | M | F | F | M | M | F | F | M |  |  |
| Medical conditions | None | None | None | None | None | None | None | None |  |  |
| Family history of T2D | None | None | None | None | None | None | None | None |  |  |
| Age (years) | 21 | 21 | 20 | 25 | 23 | 19 | 23 | 18 | **21** | 1 |
| Blood pressure systolic (mmHg) | 118 | 115 | 104 | 107 | 136 | 110 | 130 | 123 | **118** | 4 |
| Blood pressure diastolic (mmHg) | 56 | 76 | 63 | 66 | 88 | 63 | 73 | 63 | **69** | 4 |
| Heart rate (beats/minute) | 47 | 59 | 68 | 65 | 71 | 61 | 60 | 87 | **65** | 4 |
| Respiratory Rate (breaths/minute) | 18 | 18 | 20 | 20 | 16 | 20 | 20 | 16 | **19** | 1 |
| Body Temperature (F) | 97.3 | 97.9 | 97.8 | 97.9 | 96.3 | 97.6 | 97.7 | 97.9 | **97.6** | 0.2 |
| Waist Circumference (cm) | 80 | 79 | 69 | 96 | 77 | 71 | 80 | 81 | **79** | 3 |
| Hip Circumference (cm) | 88 | 86 | 92 | 99 | 100 | 79 | 87 | 103 | **92** | 3 |
| Body height (inch) | 71 | 64 | 66 | 69 | 74 | 61 | 68 | 70 | **68** | 1 |
| Body weight (lb) | 165 | 135 | 129 | 160 | 151 | 122 | 123 | 167 | **144** | 7 |
| BMI (kg/m2) ^a^ | 23.3 | 23.2 | 20.7 | 23.6 | 19.6 | 23 | 18.8 | 23.8 | **22.0** | 0.7 |
| **Blood chemistry** | | | | | | | | | | |
| HBA1c (%) ^b^ | 5.2 | 5.4 | 5 | 5.4 | 5.4 | 5.5 | 5.2 | 5.6 | **5.3** | 0.1 |
| Insulin (pmol/L) | 38 | 36 | 113 | 24 | 36 | 28 | 35 | 32 | **43** | 10 |
| Total Cholesterol (mg/dL) | 109 | 121 | 121 | 163 | 109 | 160 | 149 | 142 | **134** | 8 |
| Triglycerides (mg/dL) | 42 | 92 | 68 | 105 | 49 | 66 | 54 | 56 | **67** | 8 |
| HDL Cholesterol (mg/dL) | 40 | 53 | 48 | 46 | 40 | 53 | 53 | 46 | **47** | 2 |
| LDL Cholesterol (mg/dL) | 61 | 50 | 59 | 96 | 59 | 94 | 85 | 85 | **74** | 6 |
| **Bioimpedance** | | | | | | | | | | |
| Percent fat mass (%) | 19 | 11 | 22 | 27 | 3 | 23 | 17 | 16 | **17** | 3 |
| Basal metabolic rate (calories) | 1874 | 1714 | 1398 | 1559 | 2024 | 1330 | 1381 | 1931 | **1651** | 96 |
| Bioresistance (Ohms) | 518 | 334 | 633 | 639 | 355 | 580 | 624 | 501 | **523** | 43 |
| **OGTT** | | | | | | | | | | |
| Glucose 0 min (mg/dl) ^c^ | 80 | 93 | 84 | 76 | 84 | 88 | 86 | 85 | **85** | 2 |
| Glucose 30min (mg/dl) | 119 | 140 | 122 | 137 | 151 | 167 | 139 | 112 | **136** | 6 |
| Glucose 60min (mg/dl) | 114 | 137 | 115 | 97 | 141 | 142 | 153 | 105 | **125** | 7 |
| Glucose 90min (mg/dl) | 85 | 114 | 100 | 109 | 89 | 116 | 114 | 113 | **105** | 4 |
| Glucose 120 min (mg/dl) ^d^ | 79 | 94 | 110 | 70 | 91 | 128 | 100 | 101 | **97** | 6 |
| **Hyperinsulinemic-euglycemic clamp** | | | | | | | | | | |
| Fasting plasma glucose before clamp (mg/dl) ^c^ | 87 | 91 | 78 | 85 | 93 | 89 | 90 | 85 | **87** | 2 |
| M value (mg/kgBW/min) ^e^ | 12 | 9 | 16 | 10 | 11 | 10 | 11 | 9 | **11** | 1 |

^a^ BMI: body mass index and criterion for overweight: ≥25 kg/m2

^b^ criterion for normal HbA1c: <5.7%

^c^ criterion for normal fasting plasma glucose: <100 mg/dl

^d^ criterion for normal glucose tolerance: <140 mg/dl

^e^ the average rate of glucose infusion during last 30 min of the hyperinsulinemic-euglycemic clamp, which is an index of insulin sensitivity.

**Supplementary Table 2**. Correlation of PP2A activity (either with or without metformin treatment) with participants’ clinical characteristics.

| X (clinical characteristics) | Y (Activity) | P-Value | R | R ^2^ | Intercept | Slope |
| --- | --- | --- | --- | --- | --- | --- |
| Age (years) | Basal | 0.39 | 0.36 | 0.13 | 0.52 | 0.023 |
| Gender | Basal | 0.95 | 0.03 | 0.00 | 0.99 | 0.007 |
| Blood pressure Systolic (mmHg) | Basal | 0.09 | 0.63 | 0.40 | 0.02 | 0.008 |
| Blood pressure diastolic (mmHg) | Basal | 0.64 | 0.20 | 0.04 | 0.80 | 0.003 |
| Heart rate (beats/minute) | Basal | 0.84 | -0.09 | 0.01 | 1.07 | -0.001 |
| Respiratory Rate (breaths/minute) | Basal | 0.76 | 0.13 | 0.02 | 0.80 | 0.011 |
| Body Temperature (F) | Basal | 0.52 | -0.27 | 0.07 | 8.23 | -0.074 |
| Waist Circumference (cm) | Basal | 0.78 | 0.12 | 0.01 | 0.83 | 0.002 |
| Hip Circumference (cm) | Basal | 0.92 | -0.05 | 0.00 | 1.07 | -0.001 |
| Body height (inch) | Basal | 0.48 | 0.29 | 0.09 | 0.26 | 0.011 |
| Body weight (lb) | Basal | 0.69 | -0.17 | 0.03 | 1.19 | -0.001 |
| BMI (kg/m2) | Basal | 0.10 | -0.62 | 0.39 | 2.03 | -0.047 |
| HbA1c (%) | Basal | 0.86 | -0.08 | 0.01 | 1.31 | -0.059 |
| Insulin (pmol/L) | Basal | 0.42 | -0.33 | 0.11 | 1.07 | -0.002 |
| Total Cholesterol (mg/dL) | Basal | 0.51 | 0.27 | 0.08 | 0.75 | 0.002 |
| Triglycerides (mg/dL) | Basal | 0.23 | -0.48 | 0.23 | 1.22 | -0.003 |
| HDL Cholesterol (mg/dL) | Basal | 0.95 | 0.03 | 0.00 | 0.97 | 0.001 |
| LDL Cholesterol (mg/dL) | Basal | 0.29 | 0.43 | 0.19 | 0.74 | 0.004 |
| % Fat mass | Basal | 0.89 | -0.06 | 0.00 | 1.02 | -0.001 |
| Basal metabolic rate (calories) | Basal | 0.71 | -0.16 | 0.02 | 1.14 | 0.000 |
| Bioresistence (Ohms) | Basal | 0.44 | 0.32 | 0.10 | 0.80 | 0.000 |
| OGTT Glucose 0 min (mg/dl) | Basal | 0.58 | -0.23 | 0.05 | 1.57 | -0.007 |
| OGTT Glucose 30 min (mg/dl) | Basal | 0.65 | 0.19 | 0.04 | 0.79 | 0.002 |
| OGTT Glucose 60 min (mg/dl) | Basal | 0.24 | 0.47 | 0.22 | 0.57 | 0.003 |
| OGTT Glucose 90 min (mg/dl) | Basal | 0.95 | 0.03 | 0.00 | 0.97 | 0.000 |
| OGTT Glucose 2h (mg/dl) | Basal | 1.00 | 0.00 | 0.00 | 1.00 | 0.000 |
| Fasting plasma glucose (mg/dL) | Basal | 0.36 | 0.38 | 0.14 | -0.10 | 0.013 |
| M value (mg/kgBW/min) | Basal | 0.78 | -0.12 | 0.02 | 1.08 | -0.008 |
| Age (years) | Metformin | 0.22 | -0.49 | 0.24 | 2.95 | -0.067 |
| Gender | Metformin | 0.25 | 0.47 | 0.22 | 1.12 | 0.275 |
| Blood pressure Systolic (mmHg) | Metformin | 0.62 | 0.21 | 0.04 | 0.85 | 0.006 |
| Blood pressure diastolic (mmHg) | Metformin | 0.47 | -0.30 | 0.09 | 2.18 | -0.009 |
| **Heart rate (beats/minute)** | Metformin | 0.04 | 0.72 | 0.52 | 0.26 | 0.020 |
| Respiratory Rate (breaths/minute) | Metformin | 0.20 | -0.51 | 0.26 | 3.21 | -0.090 |
| Body Temperature (F) | Metformin | 0.58 | 0.23 | 0.05 | -11.58 | 0.134 |
| Waist Circumference (cm) | Metformin | 0.67 | 0.18 | 0.03 | 0.98 | 0.007 |
| Hip Circumference (cm) | Metformin | 0.16 | 0.55 | 0.30 | -0.40 | 0.021 |
| Body height (inch) | Metformin | 0.46 | 0.31 | 0.10 | -0.13 | 0.025 |
| Body weight (lb) | Metformin | 0.16 | 0.55 | 0.30 | 0.22 | 0.009 |
| BMI (kg/m2) | Metformin | 0.39 | 0.36 | 0.13 | 0.29 | 0.057 |
| HbA1c (%) | Metformin | 0.17 | 0.54 | 0.29 | -3.21 | 0.888 |
| Insulin (pmol/L) | Metformin | 0.59 | -0.23 | 0.05 | 1.64 | -0.002 |
| Total Cholesterol (mg/dL) | Metformin | 0.58 | 0.23 | 0.05 | 1.09 | 0.003 |
| Triglycerides (mg/dL) | Metformin | 0.54 | -0.26 | 0.07 | 1.78 | -0.004 |
| HDL Cholesterol (mg/dL) | Metformin | 0.69 | -0.17 | 0.03 | 2.00 | -0.010 |
| LDL Cholesterol (mg/dL) | Metformin | 0.34 | 0.39 | 0.15 | 1.04 | 0.007 |
| % Fat mass | Metformin | 0.91 | 0.05 | 0.00 | 1.50 | 0.002 |
| Basal metabolic rate (calories) | Metformin | 0.35 | 0.38 | 0.15 | 0.80 | 0.000 |
| Bioresistance (Ohms) | Metformin | 0.89 | 0.06 | 0.00 | 1.45 | 0.000 |
| OGTT Glucose 0 min (mg/dl) | Metformin | 0.81 | -0.10 | 0.01 | 2.08 | -0.006 |
| OGTT Glucose 30 min (mg/dl) | Metformin | 0.18 | -0.53 | 0.28 | 2.79 | -0.009 |
| OGTT Glucose 60 min (mg/dl) | Metformin | 0.28 | -0.44 | 0.19 | 2.38 | -0.007 |
| OGTT Glucose 90 min (mg/dl) | Metformin | 0.60 | 0.22 | 0.05 | 0.94 | 0.006 |
| OGTT Glucose 2h (mg/dl) | Metformin | 0.92 | 0.04 | 0.00 | 1.47 | 0.001 |
| Fasting plasma glucose (mg/dL) | Metformin | 0.59 | -0.23 | 0.05 | 2.92 | -0.016 |
| M value (mg/kgBW/min) | Metformin | 0.47 | -0.30 | 0.09 | 1.99 | -0.041 |

Supplementary method: in vitro differentiation of myotubes and metformin treatment

Once the myoblasts reached 95% confluence in 10-cm petri dishes, the growth medium was replaced with 10 ml of differentiation medium (DMEM containing 2% horse serum and 1% PSG) on the following day (designated as day 1). The cell cultures were incubated in a humidified atmosphere at 37°C and 5% CO2. On day 3, the differentiation medium was discarded, and the cells were supplemented with 10 ml of fresh differentiation medium. On day 4, the cells were treated with or without 50μM metformin for 24 hours.
