# Supplementary figures and images for: Metformin Increases Protein Phosphatase 2A Activity in Primary Human Skeletal Muscle Cells Derived from Lean Healthy Participants
Source: J Diabetes Res. 2021 Jul 28;2021:9979234. doi: 10.1155/2021/9979234 (PMC8342103; doi:10.1155/2021/9979234)

## Slide 1
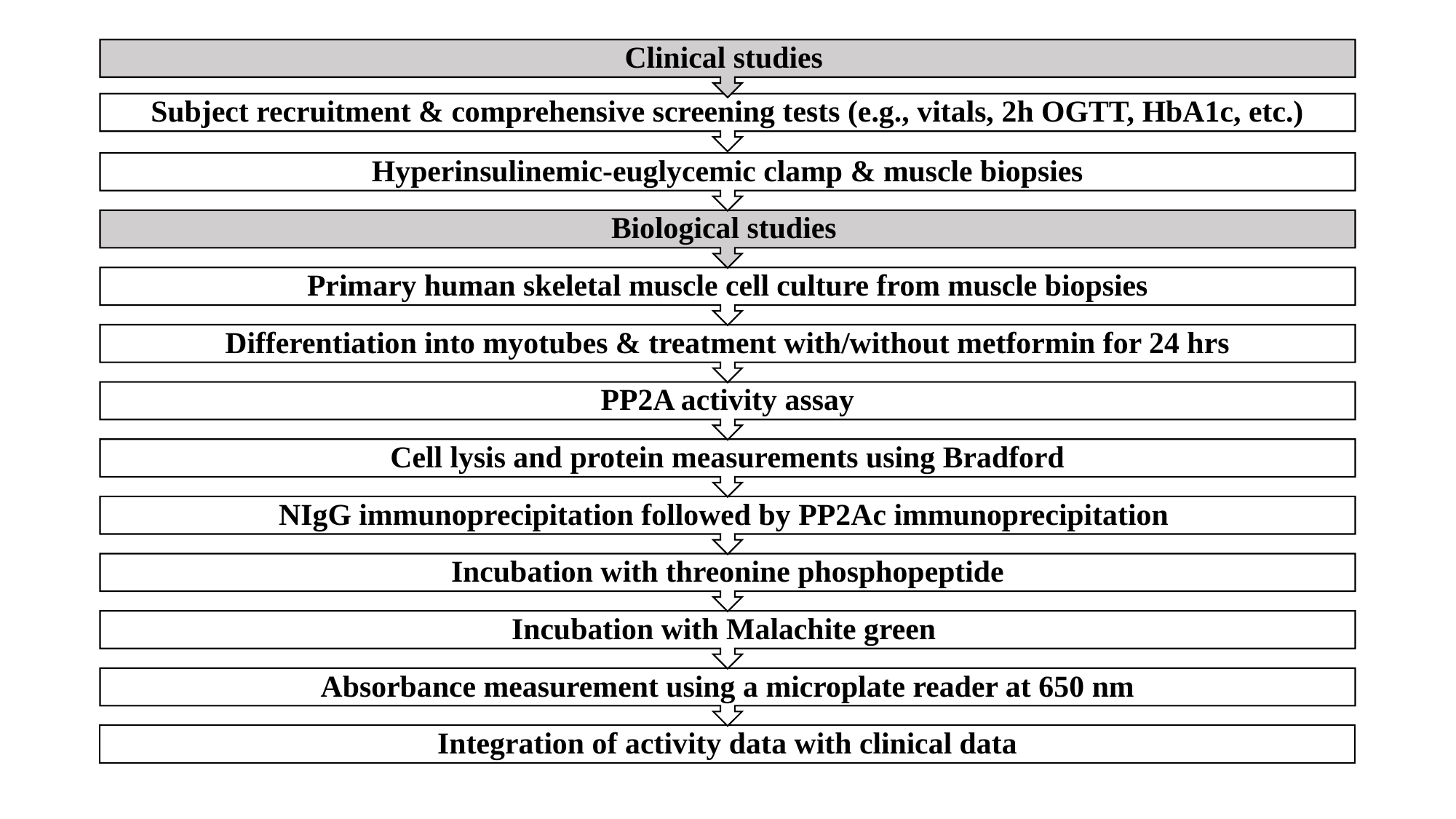

Supplement: Supplementary Materials — Supplementary information includes Supplementary Figure 1, Supplementary Table 1, and Supplementary Table 2 as well as supplementary method. Supplementary 1 Supplementary Table 1: clinical characteristics of lean nondiabetic participants in the study. All measurements were done after an overnight fast. Supplementary Table 2: correlation of PP2A activity (either with or without metformin treatment) with participants' clinical characteristics. Supplementary 2 Supplementary Figure 1: schematic diagram of clinical and biological studies. [file 9979234.f1.zip › 9979234.f1/Supplementary Figure 1.pptx]
